# Sympathetic Stimulation Upregulates the Ca^2+^ Channel Subunit, Ca_V_α2δ1, via the β1 and ERK 1/2 Pathway in Neonatal Ventricular Cardiomyocytes

**DOI:** 10.3390/cells11020188

**Published:** 2022-01-06

**Authors:** Aya Al Katat, Juan Zhao, Angelino Calderone, Lucie Parent

**Affiliations:** 1 Département de Pharmacologie et Physiologie, Faculté de Médecine, Université de Montréal, Montréal, QC H3T 1J4, Canada; a.farajatat@gmail.com (A.A.K.); Angelo.calderone@umontreal.ca (A.C.); 2 Research Center, Montreal Heart Institute, 5000 Rue Belanger, Montréal, QC H1T 1C8, Canada; Juan.Zhao@icm-mhi.org

**Keywords:** cardiac hypertrophy, L-type Ca^2+^ channels, sympathetic stimulation, adrenergic stimulation, ERK 1/2, cardiomyocytes

## Abstract

Intracellular Ca^2+^ overload secondary to chronic hemodynamic stimuli promotes the recruitment of Ca^2+^-dependent signaling implicated in cardiomyocyte hypertrophy. The present study tested the hypothesis that sympathetic-mediated hypertrophy of neonatal rat ventricular cardiomyocytes (NRVMs) translated to an increase in calcium influx secondary to the upregulation of Ca_V_1.2 channel subunits. Confocal imaging of norepinephrine (NE)-treated NRVMs revealed a hypertrophic response compared to untreated NRVMs. L-type Ca_V_1.2 peak current density was increased 4-fold following a 24-h stimulation with NE. NE-treated NRVMs exhibited a significant upregulation of Ca_V_α2δ1 and Ca_V_β3 protein levels without significant changes of Ca_V_α1C and Ca_V_β2 protein levels. Pre-treatment with the β_1_-blocker metoprolol failed to inhibit hypertrophy or Ca_V_β3 upregulation whereas Ca_V_α2δ1 protein levels were significantly reduced. NE promoted the phosphorylation of ERK 1/2, and the response was attenuated by the β_1_-blocker. U0126 pre-treatment suppressed NE-induced ERK1/2 phosphorylation but failed to attenuate hypertrophy. U0126 inhibition of ERK1/2 phosphorylation prevented NE-mediated upregulation of Ca_V_α2δ1, whereas Ca_V_β3 protein levels remained elevated. Thus, β_1_-adrenergic receptor-mediated recruitment of the ERK1/2 plays a seminal role in the upregulation of Ca_V_α2δ1 in NRVMs independent of the concomitant hypertrophic response. However, the upregulation of Ca_V_β3 protein levels may be directly dependent on the hypertrophic response of NRVMs.

## 1. Introduction

Normal intracellular Ca^2+^ cycling and homeostasis are required for cardiac excitability, contractility, and gene expression [1,2]. Several studies have reported that intracellular calcium overload secondary to a sustained hemodynamic stimulus contributed to the development of cardiac hypertrophy via recruitment of the nuclear factor of activated T cells (NFAT) pathway and calmodulin kinase-dependent signaling events [3,4,5]. Cardiac hypertrophy is an adaptive mechanism secondary to a sustained chronic hemodynamic overload [6,7,8]. In response to elevated mean arterial pressure, the heart develops a concentric pattern of cardiac hypertrophy [6,7,8]. During the hypertrophic response, new sarcomeres are added in a parallel fashion leading to an increase in the width of individual ventricular cardiomyocytes [6,7,8]. Morphologically, the latter response translates to increased ventricular wall thickness and reduced chamber diameter [6,7,8].

In ventricular cardiomyocytes, voltage-gated L-type Ca^2+^ channels (LTCCs) mediate extracellular Ca^2+^ entry initiating Ca^2+^-induced Ca^2+^ release and triggering cardiac excitation-contraction coupling [1,9,10]. LTCC are oligomeric proteins composed of the pore-forming subunit Ca_V_α1 bound to the auxiliary subunits Ca_V_β, calmodulin, and Ca_V_α2δ [11,12]. In ventricular cardiomyocytes, the major isoforms are Ca_V_α1C [13,14], Ca_V_β2 [14,15,16], and Ca_V_α2δ1 [17,18]. Ca_V_α1C is the pore-forming subunit responsible for Ca^2+^ selectivity, activation and inactivation kinetics [10,19]. Ca_V_β is a cytoplasmic chaperone protein that supports the trafficking of Ca_V_1.2 channels to the plasma membrane [20,21,22]. Ca_V_α2δ1 is a large extracellular Glycosylphosphatidylinositol (GPI)-anchored protein that facilitates channel activation at physiological voltages and stimulates (≈5-fold) peak current density [9,23,24,25]. In human embryonic kidney (HEK) cells, the co-expression of Ca_V_α1C and Ca_V_α2δ1 increased the peak current density of Ca_V_1.2 [23].

Numerous studies have identified the sympathetic system as an essential homeostatic mechanism providing inotropic support, whereas chronic activation contributes in part to the progression of cardiac hypertrophy. Acute sympathetic stimulation of ventricular cardiomyocytes via the β_1_-adrenergic receptor activation increases voltage-gated L-type Ca^2+^ currents via protein kinase A phosphorylation which releases the Rad-mediated inhibition through a likely reduction in the affinity of Rad with the membrane and the Ca_V_β-subunit of the channel [26]. The relationship between chronic stimulation of the sympathetic system and Ca^2+^ overload remains unresolved. Therefore, the present study tested the hypothesis that sympathetic-mediated hypertrophy of neonatal rat ventricular cardiomyocytes (NRVMs) translated to an increase in Ca^2+^ influx secondary to the upregulation in the protein expression of Ca_V_1.2 channel subunits.

## 2. Material and Methods

### 2.1. Animal Ethics Approval

The use and care of laboratory rats were according to the Canadian Council for Animal Care and approved by the Animal Care Committee of the Montreal Heart Institute.

### 2.2. Isolation of Neonatal Ventricular Cardiomyocytes

Neonatal rat ventricular cardiomyocytes (NRVMs) were isolated from 1-day old Sprague-Dawley rat pups (sacrificed by decapitation) (Charles River, Senneville, QC, Canada) as previously described [7,27,28]. Ventricular cells were plated at a density of 400 cells/mm^2^ in DMEM-low glucose (Hyclone Laboratories, Logan, UT, USA) supplemented with 7% heat-inactivated FBS and 1% penicillin-streptomycin for 24 h, subsequently washed and maintained in DMEM-low glucose containing insulin (5 μg/mL), transferrin (5 μg/mL), and selenium (5 ng/mL) (ITS; BD Bioscience, Bedford, MA, USA) for 24 h prior to the experimental protocol. In all experiments, neonatal rat ventricular cardiomyocytes were plated at a density of 400–500 cells/mm^2^.

### 2.3. Treatment of Neonatal Ventricular Cardiomyocytes

Cardiomyocytes were treated with 1 µM norepinephrine (NE) for 1, 4, 16, 24, or 48 h. The untreated cardiomyocytes were subjected to the same procedure and plating duration as norepinephrine-treated cardiomyocytes. In parallel experiments, neonatal cardiomyocytes were pre-treated with 10 µM U0126 (CST, 9303S, Danvers, MA, USA) or 100 nM metoprolol tartrate (TOCRIS, 3251, Barton, UK) for 30 min to 1 h prior to the addition of 1 µM NE for 24 h.

### 2.4. Immunofluorescence and Surface Area Assessment

Neonatal rat ventricular cardiomyocytes (NRVMs) were plated on glass coverslips coated with poly-D-lysine in 12-well plates. Cardiomyocytes were fixed with 2% paraformaldehyde for 20 min with 2% paraformaldehyde. Immunofluorescence was performed as previously described [27]. Primary antibodies employed include mouse anti-Troponin-T (1:200; Abcam, Cambridge, UK; ab8295), rabbit anti-Ca_V_α2δ1 (1:200, Alomone, Jerusalem, Israel, ACC-015), and rabbit anti-Ca_V_α1C (1:200, Alomone, ACC-003). Secondary antibodies employed include goat anti-mouse coupled to Alexa 555 (1:800; Invitrogen, Waltham, MA, USA; A21424) and donkey anti-rabbit coupled to Alexa 488 (1:800; Invitrogen; A21206). The nucleus was identified with 4′,6′-diamidino-2-phenylindole (DAPI, Sigma-Aldrich, St. Louis, MO, USA) staining, and 4′,6-diamidino-2-phenylindole DAPI (1:800). Images were captured by confocal microscope with 20× or 63× objective. Images were analyzed using Zeiss LSM image software. The cross-sectional area (µm^2^) of 250 Troponin-T^+^ mononucleated cardiomyocytes from 5 different images per condition was determined using Zeiss LSM image software. For the colocalization analysis, wheat germ agglutinin (WGA) antibody (1:200; Invitrogen; W32466) was added to cardiomyocytes prior to fixation.

### 2.5. Western Blot

Protein lysates of neonatal rat ventricular cells were prepared and subjected to SDS-electrophoresis, as previously described [27]. Lysates containing 30 µg of proteins were subjected to SDS-polyacrylamide gel (8%) electrophoresis and transferred to a nitrocellulose paper. Antibodies used were rabbit anti-Ca_V_α2δ1 (1:1000, Alomone ACC-015), rabbit Ca_V_α1C (1:1000, Alomone ACC-003), rabbit Ca_V_β2 (1:5000, Alomone ACC-105), rabbit Ca_V_β3 (1:500, Alomone ACC-008), rabbit Phospho-ERK 1/2 (Cell Signaling Technology, Danvers, MA, USA, 9101S), Total ERK 1/2 (Cell Signaling Technology, 137F5) and rabbit GAPDH (Jackson, West Grove, PA, USA, 111-035-144). Membranes were incubated with primary antibodies overnight at 4 °C followed by incubation with secondary anti-rabbit antibody (1:10,000, Jackson) for 2 h at room temperature. Signal was detected using enhanced chemiluminescence (ECL) substrate.

### 2.6. Patch-Clamp

Whole-cell voltage-clamp recordings were performed in isolated NRVMs. Patch-clamp experiments were carried out with the Axopatch 200-B amplifier (Molecular Devices, Union City, CA, USA). PClamp software Clampex 10.4 coupled to a Digidata 1440A acquisition system (Molecular Devices, San Jose, CA, USA) was used for on-line data acquisition and analysis. Electrodes were filled with a solution containing (in mM) 140 CsCl, 0.6 NaGTP, 3 MgATP, and 10 EGTA, 10 HEPES, titrated to pH 7.4 with KOH. Pipette resistance ranged from 3 to 5 megohms. The bath solution contained (in mM) 135 NMDG, 20 tetraethylammonium chloride, 2 CaCl_2_, 1 MgCl_2_, and 10 HEPES, titrated to pH 7.4 with HCI. The measurements were performed at room temperature (22–25 °C).

Following a 40 ms prepulse to −40 mV to inactivate Na^+^ channels, Ca^2+^ currents were elicited from a holding potential of −80 mV and were depolarized to potentials ranging from −80 to 50 mV in 5 mV increments lasting 450 ms for each step (protocol shown in the inset above the current traces). Ca^2+^ current densities (pA/pF) were obtained by dividing the peak currents by the cell capacitance. Average I-V curves were obtained by plotting peak current densities as a function of applied voltage. The I-V relationships were fitted to a Boltzmann equation. Patch-clamp data were analyzed using Clamp fit software 10.4 (Molecular Devices), Microsoft Excel 2016 (Microsoft, Redmond, WA. USA), and Origin 2020 (Northhampton, MA, US).

### 2.7. Statistics

Data were presented as the mean ± S.E.M., and n represents the number of rat litters employed. Data were evaluated by one-way ANOVA analysis followed by Dunnett’s multiple comparison test or Tukey’s multiple comparison test (GraphPad). A value of *p* < 0.05 was considered statistically significant.

## 3. Results

### 3.1. Norepinephrine Induced Neonatal Rat Ventricular Cardiomyocyte Hypertrophy and Increased Ca_V_1.2 Peak Current Density

**Figure 1:**
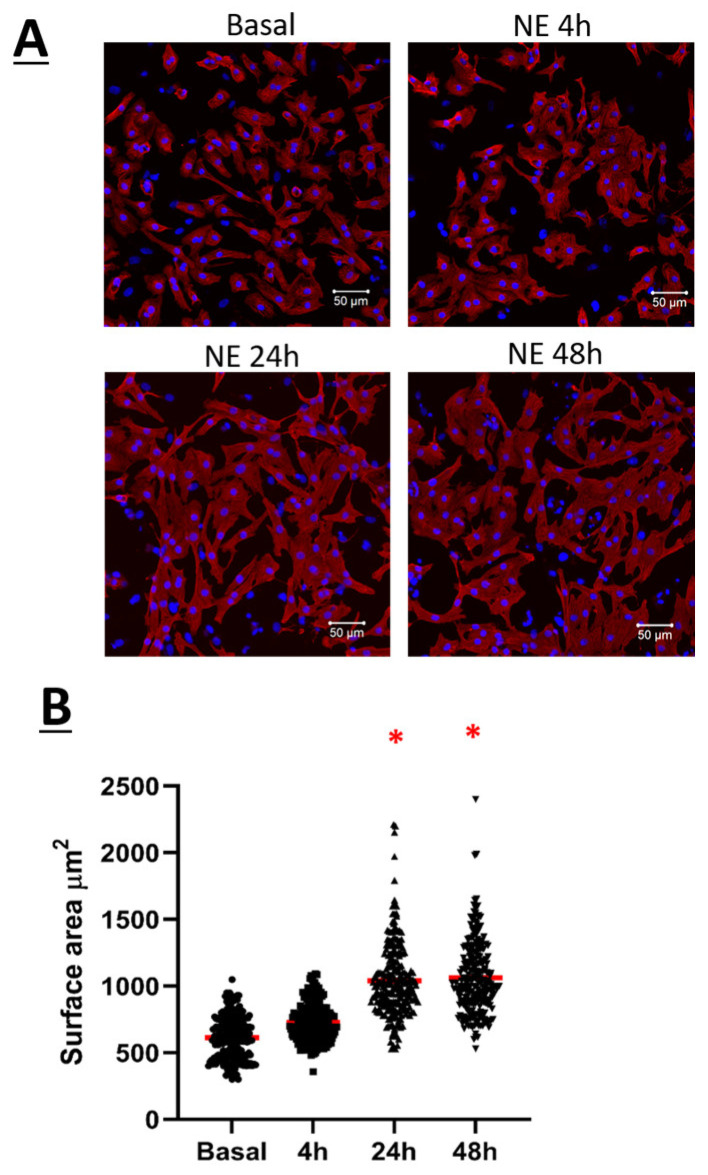
(**A**) Neonatal rat ventricular cardiomyocytes (NRVMs) in basal condition, treated with 1 µM norepinephrine (NE) for 4 h, 24 h or 48 h. Cardiomyocytes were fixed with 2% PAF for 20 min at room temperature. Cardiomyocytes were then permeabilized with 0.2% Triton-X. Primary antibody: mouse anti-Troponin-T was added for 90 min at room temperature followed by overnight incubation at 4 °C. This is followed by incubation of secondary antibody: Goat anti-mouse Alexa 555; and DAPI for 90 min at room temperature in the dark. Images were captured by microscope with 20× objective. Images were analyzed using ZEN software, and the surface area of 250 cells was measured by marking the borders of each cell. (**B**) Dot plot showing the surface area of each cardiomyocyte. The red line represents the mean surface area of 250 cells. * *p* < 0.01 vs. Basal (5 cardio preparation). Statistical analysis was performed using one-way ANOVA.

**Figure 2:**
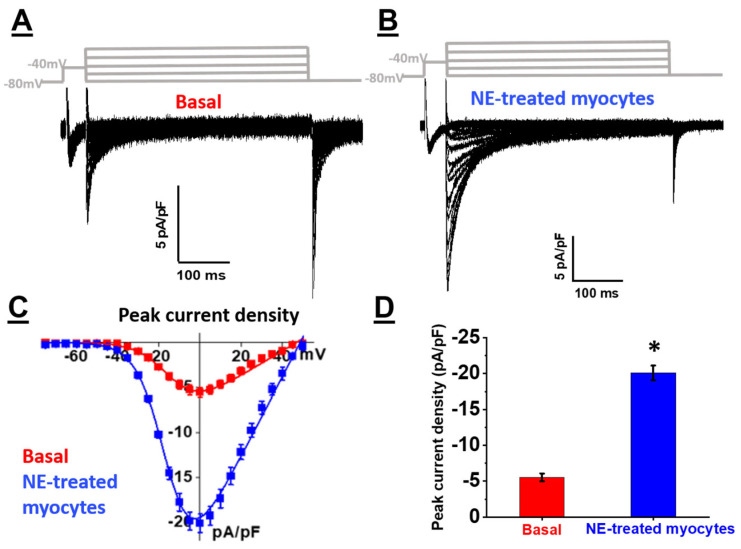
(**A**,**B**) Representative whole-cell Ca^2+^ current traces were recorded from neonatal rat cardiomyocytes under basal conditions (in red) or were treated with 1 µM norepinephrine (NE) for 24 h (in blue). In all cases, the cells were bathed in a saline physiological solution containing 2 mM Ca^2+^ in the absence of NE. The pulse protocol is shown above the traces. Currents were elicited from a holding potential of −80 mV and recorded at potentials ranging from −80 to 50 mV in 5 mV increments. Na^+^ currents were suppressed by applying a 40 ms prepulse to −40 mV as shown in the inset above the current traces. (**C**) Mean current-voltage relationships of whole-cell Ca^2+^ currents recorded from neonatal rat cardiomyocytes (NRVMs). Current densities were obtained by normalizing whole-cell current amplitudes to the membrane capacitance and were plotted versus applied voltages. The Boltzman analysis of Ca^2+^ channel activation voltage yielded a mid-point of activation at V_1/2_ = −14 ± 1 mV (mean ± S.E.) (*n* = 9, *N* = 2 repetitions) under basal conditions, and a mid-point of activation at V_1/2_ = −16 ± 1 mV (*n* = 5, *N* = 2 repetitions) for NE-treated NRVMs (*p* = 0.13). Experiments were carried out with 2 distinct cell preparations, and the total number of cells that were patched were pooled. (**D**) Bar graph of the average peak current density of basal and NE-treated groups. The NE-treated group displays a 4-fold increase in the peak current density compared with that of the basal currents (−20 ± 1 pA/pF for NE-treated vs. −5.5 ± 0.6 pA/pF for basal, * *p* < 0.01).

The temporal hypertrophic response of neonatal rat ventricular cardiomyocytes (NRVMs) in response to 1 µM norepinephrine (NE) was examined. As compared to untreated NRVMs, a significant hypertrophic response as depicted by the increase in cell surface area was detected after a 24-h stimulation with NE, and hypertrophy persisted after 48 h (Figure 1A,B). In parallel, electrophysiological recordings using the whole-cell patch-clamp technique were performed on NE-treated NRVMs to assess the Ca_V_1.2 peak current density. Whole-cell Ca^2+^ current traces were detected in untreated NRVMs (−5.5 ± 0.6 pA/pF, *n* = 9, *N* = 2) (Figure 2A,B). In hypertrophied NRVMs secondary to a 24-h stimulation with 1 µM NE, a significant (*p* < 0.01) four fold increase in peak current density was detected (−20 ± 1.0 pA/pF, *n* = 5, *N* = 2) (Figure 2A,B). By contrast, activation kinetics of Ca_V_1.2 Ca^2+^ currents in untreated and hypertrophied NRVMs were similar as the analysis of the Ca^2+^ whole-cell conductance properties yielded a V_1/2_ of −14 ± 1 mV for untreated and −16 ± 1 mV for hypertrophied NRVMs (*p* = 0.13) (Figure 2C).

### 3.2. Ca_V_α2δ1 and Ca_V_β3 Are Upregulated in Norepinephrine-Induced Hypertrophy of Neonatal Rat Ventricular Cardiomyocytes

**Figure 3:**
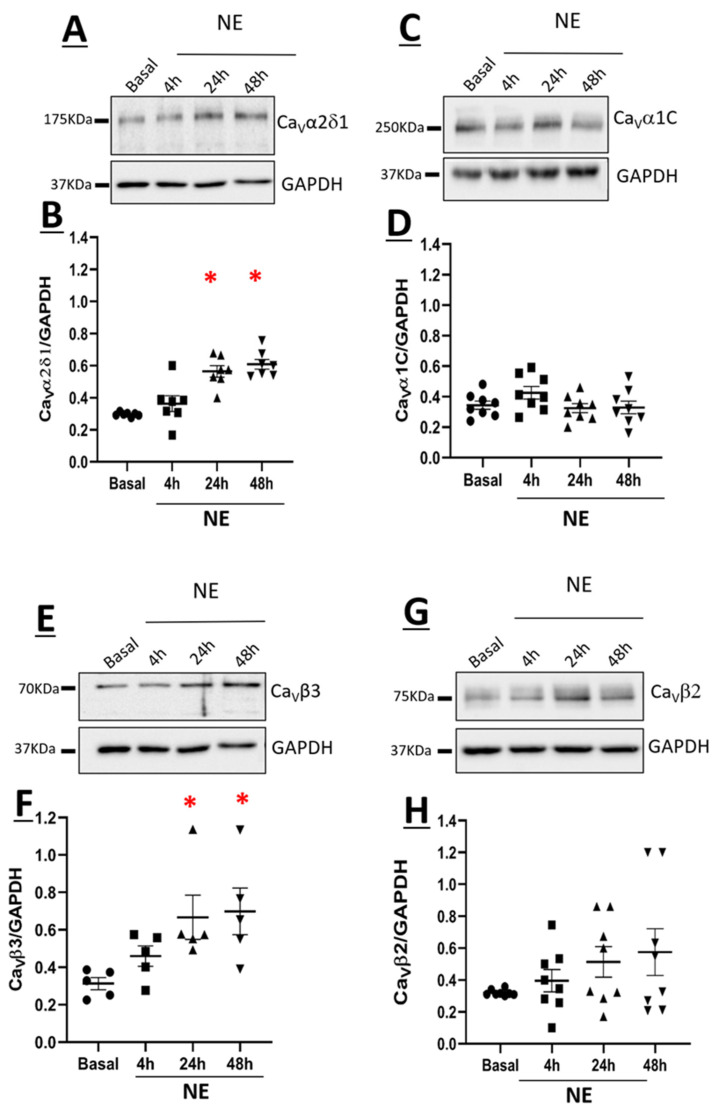
Expression of Ca_V_α2δ1 (**A**), Ca_V_α1C (**C**), Ca_V_β3 (**E**), and Ca_V_β2 (**G**) in neonatal rat cardiomyocytes. Total proteins were extracted from neonatal rat ventricular cardiomyocytes. Proteins were separated on an 8% SDS-polyacrylamide gel, transferred to a nitrocellulose membrane, and probed with anti-Ca_V_α2δ1, anti-Ca_V_α1C, anti-Ca_V_β3, anti-Ca_V_β2, and anti-GAPDH antibodies overnight and then incubated with HRP-conjugated goat anti-rabbit secondary antibody. Lanes were loaded with 30 μg of proteins. NE: cardiomyocytes treated with 1 µM norepinephrine. Graph showing the total protein expression of Ca_V_α2δ1 (**B**), Ca_V_α1C (**D**), Ca_V_β3 (**F**), and Ca_V_β2 (**H**) normalized to GAPDH. * *p* < 0.01 vs. Basal. Statistical analysis was performed using one-way ANOVA.

The stimulation of NRVMs with 1µM NE led to a significant increase of Ca_V_α2δ1 protein levels at 24 h and remained elevated at 48 h as compared to untreated NRVMs (Figure 3A,B). Furthermore, 1µM NE treatment of NRVMs for 24 and 48 h significantly increased Ca_V_β3 protein levels (Figure 3E,F). The increase of Ca_V_α2δ1 and Ca_V_β3 protein levels coincided with the hypertrophic response elicited by norepinephrine (Figure 1A,B). By contrast, Ca_V_α1C and Ca_V_β2 protein levels were not significantly altered in NRVMs treated with NE as compared to untreated NRVMs (Figure 3C,D,G,H).

### 3.3. The Subcellular Localization of Ca_V_α2δ1 or Ca_V_α1C Was Not Altered in Hypertrophic Neonatal Rat Ventricular Cardiomyocytes

**Figure 4:**
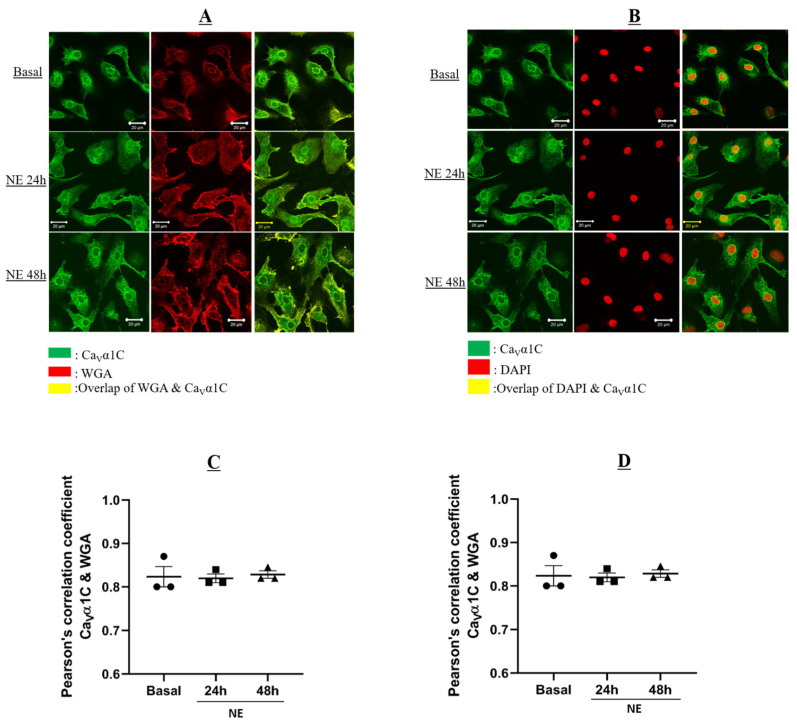
Neonatal rat cardiomyocytes in basal condition or treated with NE for 24–48 h. WGA-Alexa 647 was added to live cardiomyocytes at room temperature followed by fixation with 2% PAF for 20 min at room temperature. Cardiomyocytes were then permeabilized with 0.2% Triton-X. Primary antibody: mouse anti-Troponin-T and rabbit anti-Ca_V_a1C were added for 90 min at room temperature, followed by overnight incubation at 4 °C. This was followed by incubation of secondary antibodies: goat anti-mouse Alexa 555; Donkey anti-rabbit Alexa 488, and DAPI for 90 min at room temperature in the dark. Images were captured by microscope with 20× objective. Images were analyzed using ZEN software. (**A**) Images showing colocalization of WGA and Ca_V_α1C. Green: Ca_V_α1C; Red: WGA; Yellow: Overlap between Ca_V_α1C and WGA. (**B**) Images showing colocalization of DAPI and Ca_V_α1C. Green: Ca_V_α1C; Red: DAPI; Yellow: Overlap between Ca_V_α1C and WGA. (**C**) Graph showing Pearson’s correlation coefficient of the overlap between WGA and Ca_V_α1C. (**D**) Graph showing Pearson’s correlation coefficient of the overlap between DAPI and Ca_V_α1C. Pearson’s correlation coefficient of the overlap is determined using ImageJ.

In the present study, NE stimulation of NRVMs failed to alter the protein levels of Ca_V_α1C (Figure 4A,C). Nonetheless, additional experiments were performed to assess whether NE altered the subcellular distribution of the Ca_V_α1C subunit. Cardiac staining was used to confirm that the cells examined were cardiomyocytes (data not shown). NRVMs were stained with cardiac troponin-T (staining not shown), Ca_V_α1C subunit, wheat germ-agglutinin (WGA), or 4′,6-diamidino-2-phenylindole (DAPI; a nuclear marker). Ca_V_α1C localization was determined by Pearson’s coefficient assessing the convergence of the immunofluorescent signal between WGA/Ca_V_α1C and DAPI/Ca_V_α1C. Figure 4A depicts immunofluorescence confocal images of NRVMs co-stained with WGA (red) and Ca_V_α1C (green). In untreated NRVMs, Ca_V_α1C staining was detected predominantly on the plasma membrane and perinuclear region (Figure 4A). In NRVMs treated with 1 µM NE for 24 or 48 h, Ca_V_α1C localization at the plasma membrane was not altered (Pearson’s coefficient, r ~ 0.85) as compared to untreated NRVMs (Pearson’s coefficient, r ~ 0.83; *p* > 0.05) (Figure 4A,C).

Figure 4B depicts immunofluorescence confocal images of NRVMs co-stained with DAPI (red) and Ca_V_α1C (green). The co-staining of NRVMs with DAPI and Ca_V_α1C confirmed the perinuclear localization (convergence depicted by yellow signal) of the pore-forming subunit (Figure 4B). In NE-treated NRVMs (24 and 48 h), Ca_V_α1C localization at the perinuclear region persisted (Pearson’s coefficient, r ~ 0.26) and was similar to that observed in untreated cardiomyocytes (Pearson’s coefficient, r ~ 0.3; *p* > 0.05) (Figure 4B,D).

**Figure 5:**
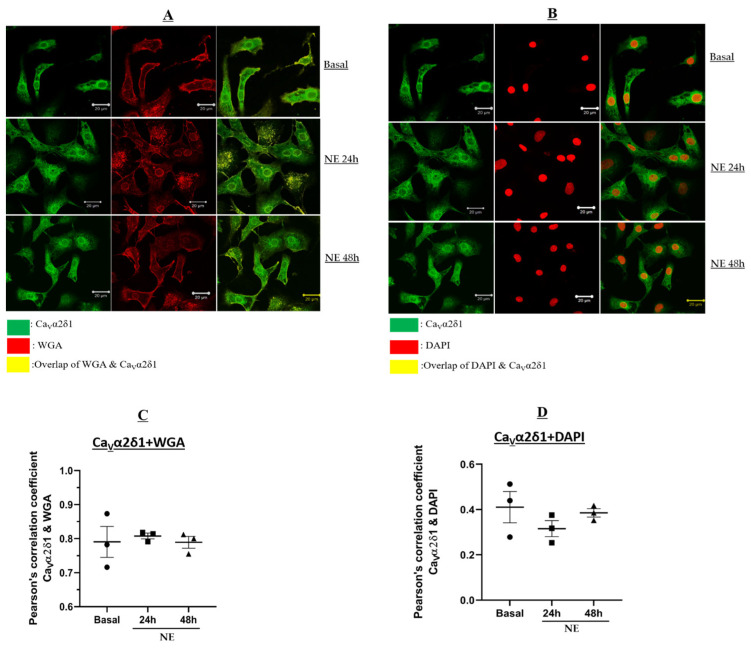
Neonatal rat cardiomyocytes in basal condition or treated with NE for 24–48 h. WGA-Alexa 647 was added to live cardiomyocytes at room temperature, followed by fixation with 2% Paraformaldehyde for 20 min at room temperature. Cardiomyocytes were then permeabilized with 0.2% Triton-X. Primary antibody: mouse anti-Troponin-T and rabbit anti-Ca_V_α2δ1 were added for 90 min at room temperature followed by overnight incubation at 4 °C. This was followed by incubation of secondary antibodies: Goat anti-mouse Alexa 555; Donkey anti-rabbit Alexa 488, and DAPI for 90 min at room temperature in the dark. Images were captured by microscope with 20× objective. Images were analyzed using ZEN software. (**A**) Images showing colocalization of WGA and Ca_V_α2δ1. Green: Ca_V_α2δ1; Red: WGA; Yellow: Overlap between Ca_V_α2δ1 and WGA. (**B**) Images showing colocalization of DAPI and Ca_V_α2δ1. Green: Ca_V_α2δ1; Red: DAPI; Yellow: Overlap between Ca_V_α2δ1 and WGA. (**C**) Graph showing Pearson’s correlation coefficient of the overlap between WGA and Ca_V_α2δ1. (**D**) Graph showing Pearson’s correlation coefficient of the overlap between DAPI and Ca_V_α2δ1. Pearson’s correlation coefficient (r) of the overlap is determined using ImageJ.

Previous studies reported that Ca_V_α2δ1 was localized at the plasma membrane of neonatal mouse cardiomyocytes [9]. Immunofluorescence experiments were performed to assess whether the upregulation of Ca_V_α2δ1 protein levels secondary to NE-induced cardiac hypertrophy was associated with a greater translocation to the plasma membrane. Therefore, the colocalization of Ca_V_α2δ1 with WGA or DAPI was examined. Figure 5A depicts immunofluorescence confocal images of NRVMs co-stained with WGA (red) and Ca_V_α2δ1 (green). In NRVMs treated with 1 µM NE for 24 or 48 h, Ca_V_α2δ1 localization at the plasma membrane was not altered (Pearson’s coefficient, r ~ 0.8) when compared to untreated NRVMs (Pearson’s coefficient, r ~ 0.79; *p* > 0.05) (Figure 5A,C). The confocal staining experiments are supportive of plasma membrane localization.

Figure 5B demonstrates immunofluorescence confocal images of NRVMs stained with DAPI (red) and Ca_V_α2δ1 (green). The co-staining of NRVMs with DAPI and Ca_V_α2δ1 in untreated NRVMs revealed a perinuclear and nuclear signal (convergence depicted by yellow signal) of the calcium subunit (Pearson’s coefficient, r ~ 0.51) (Figure 5B). As seen, Ca_V_α2δ1 localization at the plasma membrane was not altered when comparing untreated NRVMs (Pearson’s coefficient, r ~ 0.79)) with NRVMs treated with 1 µM NE for 24 or 48 h (Pearson’s coefficient, r ~ 0.8); *p* > 0.05 (Figure 5A,C). Figure 5B demonstrates immunofluorescence confocal images of NRVMs stained with DAPI (red) and Ca_V_α2δ1 (green). The co-staining with DAPI and Ca_V_α2δ1 in untreated NRVMs revealed the presence of the subunit in perinuclear and nuclear regions, depicted by a yellow signal (Pearson’s coefficient, r ~ 0.51) (Figure 5B). Ca_V_α2δ1 localization in the perinuclear and nuclear regions persisted and was non-significantly reduced in NRVMs treated with 1 µM NE for 24 h or 48 h, (Pearson’s coefficient, r ~ 0.34; *p* > 0.05; *n* = 3) (Figure 5B,D). In NRVMs treated with 1 µM NE for 24 h or 48 h, Ca_V_α2δ1 localization in the perinuclear and nuclear regions persisted and was non-significantly reduced (Pearson’s coefficient, r ~ 0.34; *p* > 0.05; *n* = 3) (Figure 5B,D).

### 3.4. β1-Adrenergic Receptor-Mediated NE-Induced Upregulation of Ca_V_α2δ1 via Recruitment of the Downstream Signaling Kinase ERK 1/2

**Figure 6:**
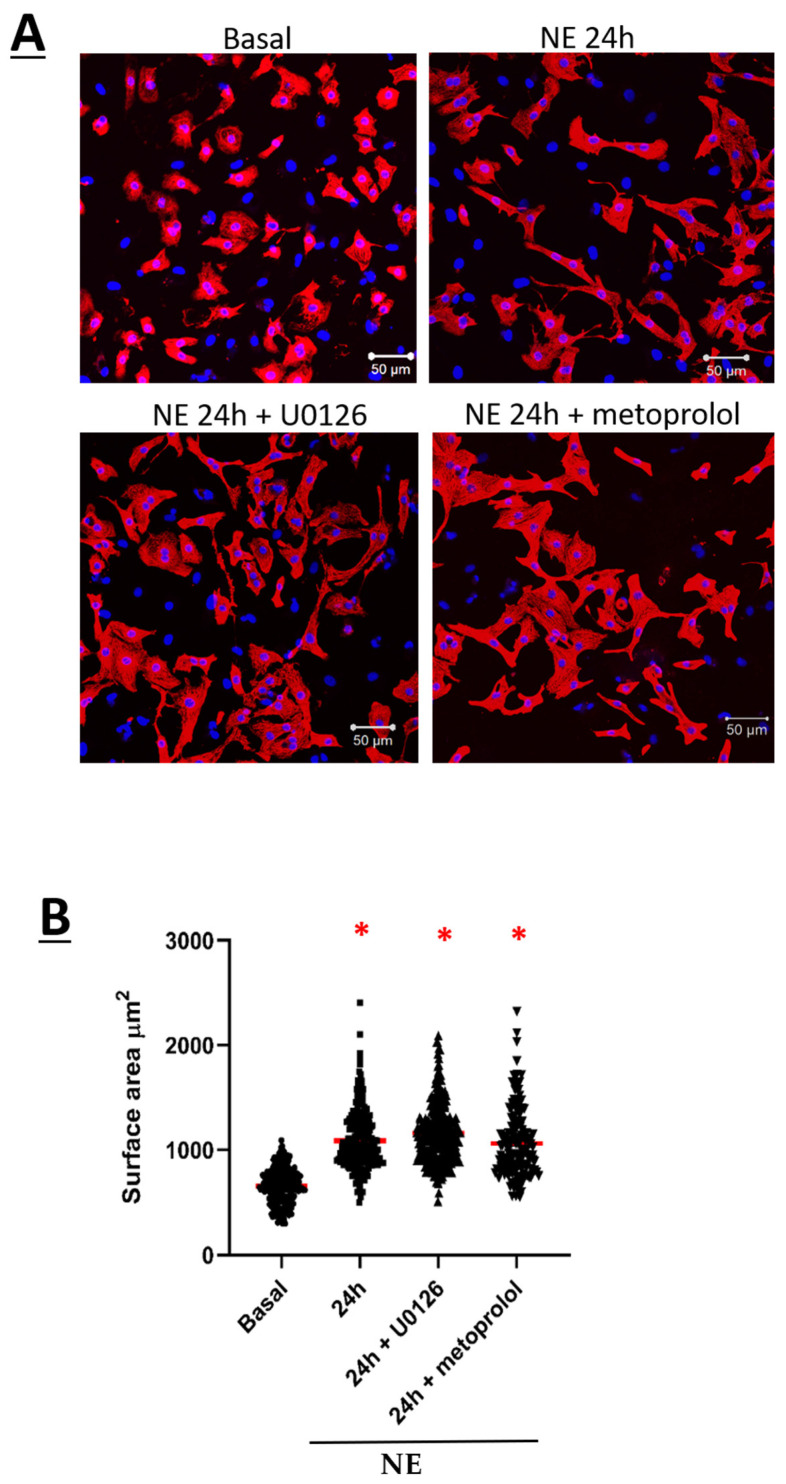
(**A**) Neonatal rat cardiomyocytes in basal condition, treated with NE for 24 h, or pre-treated with either 10 µM U0126 or metoprolol tartrate (β1-blocker) for 1 h followed by treatment with NE for 24 h. Cardiomyocytes were fixed with 2% PAF for 20 min at room temperature. Cardiomyocytes were then permeabilized with 0.2% Triton-X. Primary antibody: mouse anti-Troponin-T was added for 90 min at room temperature followed by overnight incubation at 4 °C. This is followed by incubation of secondary antibody: Goat anti-mouse Alexa 555; and DAPI for 90 min at room temperature in the dark. Images were captured by microscope with 20× objective. Images were analyzed using ZEN software, and the surface area of 250 cells was measured by marking the borders of each cell. Statistical analysis was performed using one-way ANOVA. (**B**) Dot plot showing the surface area of each cardiomyocyte. The red line represents the mean surface area of 250 cells. * *p* < 0.01 vs. Basal. (*n* = 4). Statistical analysis was performed using one-way ANOVA.

**Figure 7:**
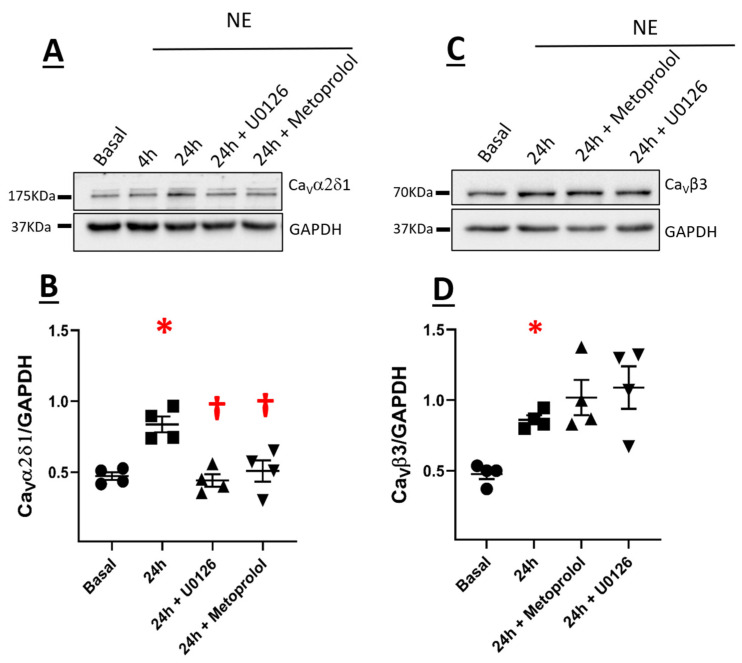
Expression of Ca_V_α2δ1 (**A**) and Ca_V_β3 (**C**) in neonatal rat cardiomyocytes. Total proteins were extracted from neonatal rat ventricular cardiomyocytes. Cells were treated with 1 µM Norepinephrine (NE) for 24 h or pre-treated with metoprolol tartrate (β1-blocker) for 1 h followed by treatment with NE for 24 h. Proteins were separated on an 8% SDS-polyacrylamide gel, transferred to a nitrocellulose membrane, and probed with anti-Ca_V_α2δ1, anti-Ca_V_β3, and anti-GAPDH antibodies overnight and then incubated with HRP-conjugated goat anti-rabbit secondary antibody. Lanes were loaded with 30 μg of proteins. NE: cardiomyocytes treated with 1 µM norepinephrine. 24 h + metoprolol tartrate: Cardiomyocytes pre-treated with metoprolol tartrate (β1-blocker) for 1 h, followed by treatment with NE for 24 h. (**B**) Graph showing the total protein expression of Ca_V_α2δ1 normalized to GAPDH. * *p* < 0.01 vs. Basal untreated NRVMs; † *p* <0.01 vs. NE 24 h. (4 cardio-preparations). (D) Graph showing the total protein expression of Ca_V_β3 normalized to GAPDH. * *p* < 0.01 vs. Basal untreated NRVMs (4 cardio-preparations). Statistical analysis was performed using one-way ANOVA.

The pre-treatment of NRVMs with the selective β_1_-adrenergic receptor antagonist metoprolol (100 nM) failed to inhibit NE-mediated hypertrophy (Figure 6A,B). However, NE-mediated upregulation of the Ca_V_α2δ1 subunit in NRVMs after a 24-h stimulation was significantly attenuated following the pre-treatment with metoprolol (Figure 7A,B). By contrast, metoprolol pre-treatment did not inhibit NE-mediated upregulation of Ca_V_β3 subunits (Figure 7C,D).

**Figure 8:**
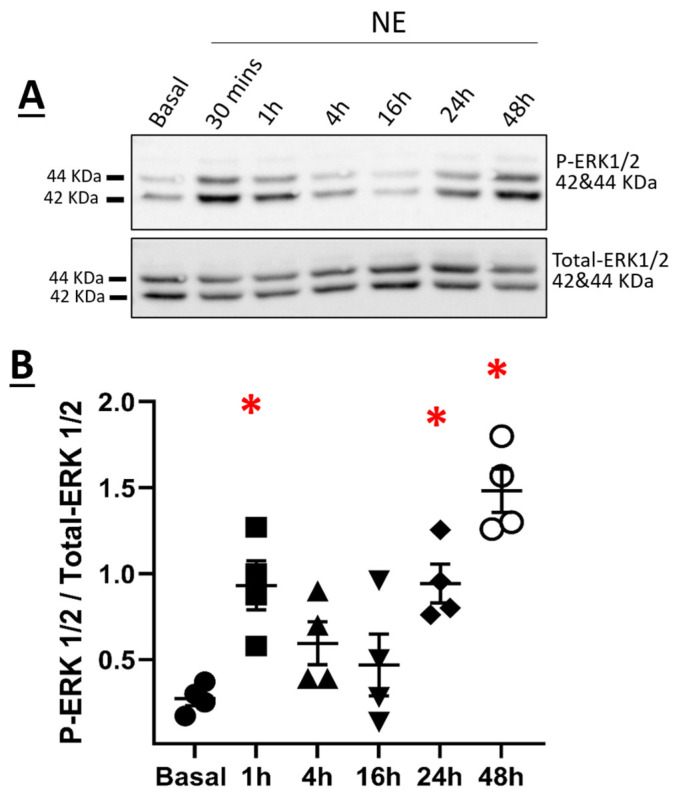
(**A**) Expression of *p*-ERK 1/2 in neonatal rat cardiomyocytes. Total proteins were extracted from neonatal rat ventricular cardiomyocytes. Proteins were separated on an 8% SDS-polyacrylamide gel, transferred to a nitrocellulose membrane, and probed with anti-P-ERK1/2 and anti-total-ERK antibodies overnight, and then incubated with HRP-conjugated goat anti-rabbit secondary antibody. Lanes were loaded with 30 μg of proteins. NE: cardiomyocytes treated with 1 µM norepinephrine. (**B**) Graph showing the total protein expression of P-ERK 1/2 normalized to total-ERK 1/2. * *p* < 0.01 vs. Basal; * *p* < 0.01 vs. Basal; (4 cardio-preparations). Statistical analysis was performed using one-way ANOVA.

**Figure 9:**
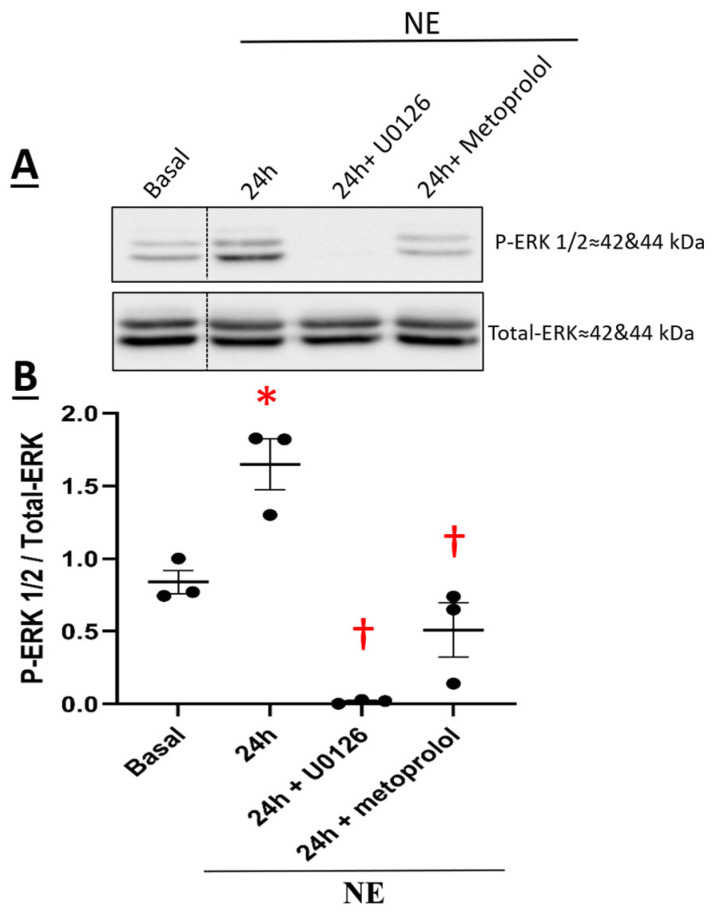
(**A**) Expression of P-ERK 1/2 in neonatal rat cardiomyocytes. Total proteins were extracted from neonatal rat ventricular cardiomyocytes. Cells were treated with 1 µM Norepinephrine (NE) for 4–24 h or pre-treated with either U0126 (P-ERK1/2 inhibitor) or metoprolol tartrate (β1-blocker) for 1 h followed by treatment with NE for 24 h. Proteins were separated on an 8% SDS-polyacrylamide gel, transferred to a nitrocellulose membrane, and probed with anti-P-ERK, anti-total-ERK antibodies overnight and then incubated with HRP-conjugated goat anti-rabbit secondary antibody. Lanes were loaded with 30 μg of proteins. NE: cardiomyocytes treated with 1 µM norepinephrine, 24 h + U0126: Cardiomyocytes pre-treated with U0126 for 1 h followed by treatment with NE for 24 h. 24 h + β1-blocker: Cardiomyocytes pre-treated with metoprolol tartrate (β1-blocker) for 1 h followed by treatment with NE for 24 h. (**B**) Graph showing the total protein expression of P-ERK normalized to total-ERK. * *p* < 0.01 vs. Basal; † *p* < 0.01 vs. NE 24 h. (3 cardio-preparations). Statistical analysis was performed using one-way ANOVA.

The temporal pattern of ERK1/2 activation in NRVMs in response to NE was examined. A transient pattern of phosphorylation was observed as ERK1/2 phosphorylation was significantly increased 1 h after NE treatment (Figure 8A,B). Thereafter, ERK1/2 phosphorylation returned to baseline levels 4 and 16 h after NE treatment (Figure 8A,B). However, in hypertrophic NRVMs, ERK1/2 phosphorylation was significantly increased 24 h after NE treatment and remained elevated at 48 h (Figure 8A,B). In the presence of the β_1_-adrenergic receptor antagonist metoprolol (100 nM), NE-mediated ERK1/2 phosphorylation was inhibited (Figure 9A,B). The pre-treatment with 10 µM U0126, a selective inhibitor of MEK1/2 (upstream activator of ERK1/2), suppressed NE-mediated phosphorylation of ERK1/2 (Figure 9A,B) but failed to attenuate the hypertrophic response (Figure 6A,B). Moreover, the pre-treatment with U0126 attenuated NE-mediated upregulation of Ca_V_α2δ1, whereas Ca_V_β3 protein levels remained elevated (Figure 7A,B).

## 4. Discussion

Numerous in vitro and in vivo studies have delineated the role of calcium-dependent signaling events linking various stimuli to ventricular cardiomyocyte hypertrophy [29,30,31]. However, the relationship between increased intracellular Ca^2+^ and the expression of the voltage-gated L-type Ca^2+^ channels in response to a hypertrophic stimulus remains unresolved. To address the latter paradigm, neonatal rat ventricular cardiomyocytes (NRVMs) were treated with norepinephrine as numerous studies have previously established a hypertrophic role of the sympathetic neurotransmitter. As demonstrated in previous studies, the exposure of NRVMs to sympathetic stimulation for 24 and 48 h led to a significant increase in the cell surface area as compared to untreated NRVMs. In parallel, L-type Ca_V_1.2 peak current density was significantly elevated in NRVMs secondary to NE-induced cardiomyocyte hypertrophy. These data provided the impetus to assess the individual role of the subunits forming the oligomeric Ca_V_1.2 channel in the increased Ca^2+^ influx in NE-induced hypertrophied NRVMs.

Activation of voltage-gated L-type Ca^2+^ channels in ventricular cardiomyocytes in response to acute sympathetic stimulation occurs predominantly via β-adrenergic receptor-mediated recruitment of protein kinase A and subsequent channel phosphorylation [32]. The present study further revealed that sympathetic system stimulation of NRVMs for a period of 24 h significantly increased voltage-gated L-type Ca^2+^ channel activity. The increase in the L-type Ca_V_1.2 peak current density in NE-treated NRVMs was associated with a significant upregulation of Ca_V_α2δ1 with a more modest increase in the protein expression of Ca_V_β3. In contrast, the protein levels of the pore-forming Ca_V_α1C and the accessory Ca_V_β2 were unchanged. Previous work from our lab revealed that co-expression of Ca_V_α1C and Ca_V_α2δ1 subunits upregulated by 5- to 10-fold the peak current density and facilitated the opening of the L-type Ca_V_1.2 activity at physiological voltages [9]. The more modest increase in Ca_V_β3 protein levels in NE-induced hypertrophy of NRVMs was reported to be insufficient to promote on its own a change in the activity of Ca_V_1.2 [9]. The upregulation of Ca_V_α2δ1 protein levels in NE-treated NRVMs was blunted by pre-treatment with the selective β_1_-blocker metoprolol, whereas Ca_V_β3 subunit upregulation remained unchanged. Moreover, metoprolol did not inhibit NE-mediated hypertrophy, which was in part consistent with the predominant role of the α_1_-adrenergic receptor in cardiomyocyte hypertrophy [33]. Collectively, these data highlight the novel finding that β_1_-adrenergic receptor-mediated upregulation of Ca_V_α2δ1 protein levels in response to NE may have contributed in part to increased L-type Ca^2+^ channel activity independent of the concomitant hypertrophic response. By contrast, upregulation of Ca_V_β3 protein levels may be directly dependent on the hypertrophic response of NRVMs secondary to sympathetic stimulation. These data are supportive of β1-adrenergic receptor-mediated NE-induced upregulation of Ca_V_α2δ1, but other regulators of adrenergic receptors need to be added to support the conclusion.

The upregulation of the Ca_V_α2δ1 protein expression was not accompanied by a significant change in its plasma membrane localization. Immunofluorescence confocal images of untreated and NE-treated NRVMs co-stained with WGA and Ca_V_α2δ1 revealed that localization of the subunit at the plasma membrane was similar. Moreover, the co-staining of DAPI and Ca_V_α2δ1 in perinuclear and nuclear regions was not significantly modified in NE-treated NRVMs. Similarly, we failed to observe any significant change in the localization of Ca_V_α1C at the plasma membrane in NE-hypertrophied NRVMs. It remains to be seen if the perinuclear/nuclear distribution of Ca_V_α2δ1 and Ca_V_α1C is exclusive to NRVMs or is conserved in adult cardiomyocytes after postnatal development.

A previous study reported that EGF stimulation of GH3 pituitary cells increased Ca_V_α2δ1 protein levels via recruitment of the ras/MEK/ERK1/2 signaling pathway [34]. In NRVMs, sympathetic stimulation of the β_1_-adrenergic receptor is known to promote ERK1/2 phosphorylation [35]. NE treatment of NRVMs translated to a biphasic pattern of ERK1/2 phosphorylation and was elevated at 24 h coincident with cardiac hypertrophy. The β_1_-adrenergic receptor antagonist metoprolol suppressed NE-mediated ERK1/2 phosphorylation. Furthermore, pharmacological inhibition of the upstream activator MEK with U0126 suppressed NE-mediated ERK1/2 phosphorylation and the concomitant upregulation of Ca_V_α2δ1 protein levels. By contrast, U0126 failed to inhibit NE-mediated NRVM hypertrophy and upregulation of Ca_V_β3 protein levels. The absence of an anti-hypertrophic effect of U0126 after NE treatment of NRVMs was consistent with previous data demonstrating that recruitment of the ERK1/2 signaling pathway alone was insufficient to promote cardiomyocyte hypertrophy in response to various stimuli [36].

The present study has revealed that in addition to the acute increase in Ca^2+^ influx via activation of the L-type Ca_V_1.2 channel after sympathetic discharge [10], chronic NE treatment of NRVMs translated to a sustained increase in Ca^2+^ channel activity. The latter response required β_1_-adrenergic receptor-mediated recruitment of the tyrosine kinase ERK1/2 translating to the increased expression of the Ca_V_1.2 auxiliary subunit, Ca_V_α2δ1. β_1_-adrenergic receptor-mediated upregulation of the Ca_V_α2δ1 subunit was independent of the hypertrophic response. Upregulation in the protein expression of the Ca_V_α2δ1 subunit secondary to sympathetic hyperactivity may hence contribute to intracellular Ca^2+^ overload with or without hypertrophy [37].

## Data Availability

The datasets used and/or analyzed during the study are available from the first and the corresponding author upon reasonable request.
